# Protein intake in children and growth and risk of overweight or obesity: A systematic review and meta-analysis

**DOI:** 10.29219/fnr.v66.8242

**Published:** 2022-02-21

**Authors:** Erik Kristoffer Arnesen, Birna Thorisdottir, Christel Lamberg-Allardt, Linnea Bärebring, Bright Nwaru, Jutta Dierkes, Alfons Ramel, Agneta Åkesson

**Affiliations:** 1 Department of Nutrition, Institute of Basic Medical Sciences, University of Oslo, Oslo, Norway; 2 Faculty of Sociology, Anthropology and Folkloristics and Health Science Institute, University of Iceland, Reykjavik, Iceland; 3 Department of Food and Nutrition, University of Helsinki, Helsinki, Finland; 4 Department of Internal Medicine and Clinical Nutrition, Institute of Medicine, Sahlgrenska Academy, University of Gothenburg, Gothenburg, Sweden; 5 Krefting Research Centre, Institute of Medicine, University of Gothenburg, Sweden; 6 Centre for Nutrition, Department of Clinical Medicine, University of Bergen, Bergen, Norway; 7 Faculty of Food Science and Nutrition, University of Iceland, Reykjavik, Iceland; 8 Unit of Cardiovascular and Nutritional Epidemiology, Institute of Environmental Medicine, Karolinska Institutet, Stockholm, Sweden

**Keywords:** dietary protein, early life nutrition, infant feeding, growth, BMI, overweight, obesity, metabolic programming, dietary guidelines, systematic review

## Abstract

**Objectives:**

The aim of this study was to examine the evidence for an association between the dietary protein intake in children and the growth and risk of overweight or obesity up to 18 years of age in settings relevant for the Nordic countries.

**Methods:**

We searched MEDLINE, Embase, Cochrane Central Register of Controlled Trials, and Scopus up to February 26, 2021 for randomized controlled trials (RCTs) or prospective cohort studies assessing for protein intake from foods (total and from different sources) in children. The outcomes include weight, height/length, adiposity indices, and/or risk of overweight and/or obesity. The risk of bias was evaluated with instruments for each respective design (Cochrane’s Risk of Bias 2.0 and RoB-NObS). A meta-analysis of five cohort studies was performed. The evidence was classified according to the criteria of the World Cancer Research Fund.

**Results:**

The literature search resulted in 9,132 abstracts, of which 55 papers were identified as potentially relevant. In total, 21 studies from 27 publications were included, of which five were RCTs and 16 were cohort studies. The RCTs found generally null effects of high-protein intake in infants on weight gain, nor that lower protein diets negatively affected growth. All included RCTs had some concern regarding the risk of bias and were limited by small sample sizes. Total protein intake and BMI were assessed in 12 cohorts, of which 11 found positive associations. The meta-analysis revealed a pooled effect estimate of 0.06 (95% CI 0.03, 0.1) kg/m^2^ BMI per one E% increment in total protein (*I*^2^ = 15.5). Therefore, the evidence for a positive relationship between total protein intake and BMI was considered *probable*. Furthermore, there was *probable* evidence for an association between higher intake of animal protein and increased BMI. There was *limited, suggestive* evidence for an effect of total protein intake and higher risk of overweight and/or obesity, while no conclusions could be made on the associations between animal vs. plant protein intake and risk of overweight and/or obesity.

**Discussion:**

In healthy, well-nourished children of Western populations, there is probably a causal relationship between a high-protein intake in early childhood (≤ 18 months) – particularly protein of animal origin – and higher BMI later in childhood, with consistent findings across cohort studies. A lack of RCTs precluded a stronger grading of the evidence.

## Popular scientific summary

- High-protein diet in infancy is suggested as a risk factor for childhood overweight and obesity.
- A systematic review of randomized controlled trials and prospective cohort studies was performed to assess the evidence of associations between dietary protein and growth, adiposity, and overweight and/or obesity in children.
- There is probable evidence for a cause-and-effect association between higher total and animal protein intake and higher BMI in children up to 18 years of age.

The prevalence rate of overweight and obesity among children has increased dramatically worldwide and constitutes a major global public health problem (1). This rate seems to have plateaued in recent years in the Nordic countries, but is still high (1, 2). Primary prevention of overweight and obesity includes the restriction of energy intake by promoting healthier food choices, with focus on fats and sugars, and an increase in energy expenditure with more physical activity. Ensuring an optimal intake of protein in childhood may be another opportunity for primary prevention. This may be especially relevant in the Nordic setting, where protein intake is commonly higher than the recommended intake (3).

Although an adequate amount and quality of dietary protein is crucial for growth, a very high-protein diet in infancy may accelerate growth and increase the likelihood of overweight and obesity in childhood and later in life (4–8). A systematic review of the Nordic Nutrition Recommendations (NNRs) for 2012 revealed that there was convincing evidence for an effect of higher protein intake in infancy and early childhood on increased growth and higher body mass index (BMI) later in childhood (5). In addition, the systematic review found suggestive evidence that intake of animal protein, especially from dairy products, was more strongly associated with growth than that of vegetable protein (5, 9).

In updating the NNR for 2022, protein intake in children in relation to growth and obesity was a prioritized subject for a systematic review by the NNR Committee. Criteria for shortlisting topics were published in 2020 (10). Briefly, it was deemed justified to perform a new systematic review if there were important new scientific data since NNR 2012 or substantial health concerns for Nordic or Baltic countries, and no recent, relevant and qualified systematic review was available on the topic (11). A scoping review by the NNR Committee identified new data since 2011 that were considered to have the potential to change the dietary reference values or food-based dietary guidance related to protein intake or animal versus plant-based protein in relation to growth and overweight in children.

The aim of this systematic review was to examine the evidence for an association between total dietary protein intake or protein from different food sources (animal and plant) in children 4 months to 5 years of age, and growth and risk of childhood overweight or obesity.

## Methods

The methodology for the present systematic review followed the guidelines for systematic reviews developed for the NNR 2022 (12, 13) and the Preferred Reporting Items for Systematic reviews and Meta-Analyses (14, 15). A protocol was pre-registered online on PROSPERO (https://www.crd.york.ac.uk/prospero) with review ID CRD42021233197. A focused research question was developed by the NNR 2022 Committee, defining the population/participants, intervention/exposure, control, outcome, timeframe, study design and settings (PI/ECOTSS), in an iterative process with the review authors. The funding source for NNR 2022 was the Nordic Council of Ministers and governmental food and health authorities of Norway, Finland, Sweden, Denmark, and Iceland (10).

### Eligibility criteria

The inclusion and exclusion criteria are outlined in the PI/ECOTSS statement (Table 1). We included original research articles with a prospective cohort design or randomized controlled trials (RCTs) on infants and children between 4 months and 5 years of age at the time of exposure. The exposure comprised reported dietary protein intake (not formula alone), and/or different protein sources, expressed as percentage of energy (E%), grams/day, or grams/kg body weight (BW). The minimum follow-up or intervention period was 4 weeks for children under 12 months of age and 3 months for ages 1–5 years. The following outcomes measured in children up to 18 years of age were considered: 1) BW (kg or z-scores/SDS, i.e. adjusted for age and sex), length/height (cm or z-scores/SDS), BMI (absolute or z-scores/SDS); 2) body composition indices (e.g. fat mass/fat mass index); or 3) risk of overweight and/or obesity.

**Table 1:** Eligibility criteria for population or participants, intervention or exposure, control, outcome, timeframe, study design and settings

Population	Intervention or exposure	Comparators	Outcomes	Timing	Setting	Study design
Children. Exposure from 4 months to 5 years of age, outcomes may be later ages.	1) Total protein intake (% of energy, grams or grams/kg body weight) 2) Amount of different dietary protein sources of animals vs. plants (% of energy, grams or grams/kg body weight). Main dietary protein sources (% of energy, grams or grams/kg body weight).	Highest versus lowest protein intake, for example, defined by quartiles, or risk difference per gram protein from one source relative to other sources. Comparison of various protein intake in randomized controlled trials (RCTs).	1) Growth or anthropometric outcomes; weight (kg or z-scores/standardized score), length/height (cm or z-scores/SDS), body mass index/isoBMI (absolute or z-scores). 2) Risk of overweight and/or obesity. 3) Body composition indices (e.g. fat-free mass and fat mass).	Intake in children <5 years of age, preferably divided in age groups (6–12, 12–24, >24 months).	Relevant for Nordic settings (excludes, e.g., populations with a high prevalence of childhood malnutrition)	RCTs, prospective cohorts (including nested case control and case-cohort studies). Study duration: intervention ≥4 weeks if <1 years, ≥3 months for 1–5 years of age.

We excluded studies assessing formula alone, studies including exclusively pre-term born infants, infants with a very low or high birthweight, infants who were malnourished, had possible growth retardation, or were from settings otherwise not relevant for the Nordic/Baltic population.

### Information sources and search strategy

A comprehensive literature search of MEDLINE (Ovid), Embase (Ovid), Cochrane Central Register of Controlled Trials, and Scopus was performed by research librarians at the medical library in the University of Oslo, Oslo, Norway up to February 26, 2021.The search strategy (Table 2 and the online supplement) was developed in collaboration with the authors, led by E.A. and B.T. and was peer-reviewed by research librarians at the Karolinska Institute in Stockholm, Sweden. There were no date or language limitations in the search strategy. Grey literature searches were not performed.

**Table 2:** Documentation of literature search

Database	Number of retrieved references
MEDLINE (Ovid)	3,963
Embase (Ovid)	5,355
Cochrane Central Register of controlled Trials	1,358
Scopus	6,805
Number of references before deduplication	17,481
Number of references after deduplication	9,132

### Selection and data collection process

Two investigators (L.B. and C.L.A.) independently reviewed titles, abstracts, and full-text articles for inclusions according to the PI/ECOTSS statement (Table 1), first in a pilot test for 10% of the titles and abstracts, using the web tool Rayyan (https://rayyan.qcri.org) in a blind mode. Potentially eligible papers were retrieved and read in full text by the same two reviewers. Disagreements about inclusion were resolved by discussion or by a third reviewer (A.Å.).

Another two authors (B.N. and J.D.) independently extracted data from the included studies into pre-specified Excel forms. The two forms were merged, and mistakes or disagreements solved also involving a third reviewer (E.A.). All results compatible with each outcome domain listed above in each study were extracted. Among the variables extracted were study design, information on recruitment, dietary intake, interventions and controls, assessment of outcomes, follow-up, drop-out, and confounders.

### Study risk of bias assessment

Two reviewers (E.A. and A.R.) independently assessed the risk of bias in each included paper, using, for randomized trials, Cochrane’s Risk of bias 2.0 tool (16), and for prospective cohort studies, the ‘Risk of Bias for Nutrition Observational Studies’ (RoB-NObS) developed by the US Department of Agriculture (USDA’s) Nutrition Evidence Systematic Review (NESR) (partly based on the Risk of Bias in Non-randomized Studies of Interventions [ROBINS-I] instrument [17–19]).

### Synthesis methods

Studies were scrutinized for synthesis eligibility per outcome, according to the PI/ECOTSS statement. The evidence was synthesized qualitatively, in which the characteristics and context of the included studies, their strengths and limitations, heterogeneity (in study characteristics and results), and relevance were reviewed and described. The main results for each outcome are listed in evidence tables. We used mean differences or regression coefficients for the anthropometric outcomes, while odds ratio (OR) was used for studies assessing the risk of overweight and/or obesity. In studies reporting repeated outcome assessments over time, the synthesis focused on the last assessment. If a study measured protein intake at multiple time points, we assessed the findings for intake after 4 months of age.

In line with the protocol, meta-analyses were only performed when more than three independent RCTs or five cohort studies with sufficient homogenous data existed. The meta-analysis approach followed recommendations from the Agency for Healthcare Research and Quality (AHRQ) and the Cochrane Handbook (20–22), and was performed with the ‘meta’ command in Stata v17. Meta-analyses were performed using a random-effects model. Potential heterogeneity between studies was quantified using the I^2^ statistic, which estimates (range 0–100%) the proportion of variance in the pooled estimates attributable to differences in estimates between studies included in the meta-analyses. As only a small number of studies (less than 10 studies) were included in the meta-analysis, evidence of publication bias or small-study effects in meta-analyses was not explored to avoid chance findings (23).

### Certainty assessment

The strength of evidence was categorized according to the World Cancer Research Fund’s grading: ‘convincing’, ‘probable’, ‘limited – suggestive’, ‘limited – no conclusion’ and ‘substantial effects unlikely’ (10). This evaluation considered the quality (risk of bias), quantity, consistency, and precision in the body of evidence.

According to this classification, the body of evidence is judged as *convincing*, that is, strong enough to support a causal relationship or lack of a relationship (12), when several conditions are met, one of which is evidence from more than one study type. The evidence is considered as *probable* when it is strong enough to support that there is a probable causal relationship and must also meet several conditions, that is, there is evidence from at least two independent cohort studies, no unexplained heterogeneity between- or within-study types, good-quality studies to confidentially exclude the possible random or systematic errors, and evidence for biological plausibility. The evidence is considered *limited – suggestive* when there is evidence from at least two independent cohort studies, a consistent direction of effect, and evidence for biological plausibility. If the evidence is so limited that no firm conclusion can be made, it is considered *limited – no conclusion*. Finally, if evidence is strong enough to support that there is a convincing absence of a causal relationship, it is considered as *substantial effects unlikely*.

## Results

### Study selection search results

**Fig. 1:**
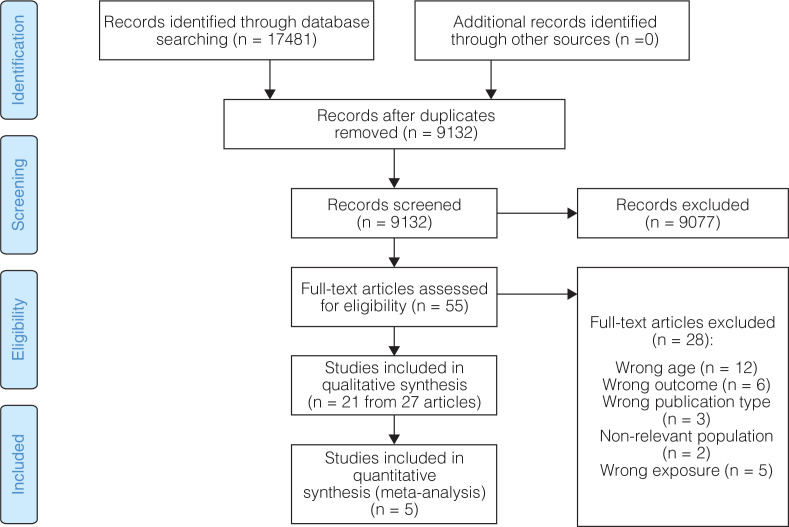
Study selection flowchart.

The results from the literature search, screening and the number of papers or studies excluded (including the reasons), as well as the studies retrieved and included in the systematic review are presented, as shown in Figure 1. The potentially eligible studies excluded after full-text assessment are listed in the online supplement.

### Study characteristics

In total, 27 publications from 21 studies were included, as outlined in Table 3, of these five (quasi) were RCTs (24–29), including 38-94 children each (total *n* = 315). All RCTs had a parallel design and were conducted in Sweden (24), Denmark (26), and the United States (25, 27–29).

**Table 3:** Selected characteristics of the included studies

Author, year (ordered by study design and alphabetical order of first author)	Country	Design	Treatment/exposures	Dietary assessment methods	Participants, N	Age at inclusion/start of intervention	Follow-up time	Type of outcomes	Confounders adjusted for
Krebs, 2006 (25)	USA	Randomized controlled trial (RCT)	Meat (10.7 g protein/day) vs. cereal (3.3 g protein/day) as exclusive complementary foods for maximum 2 months, and then allowed to include more types.	Food records	88	5 months (most started at 6 months of age)	7 months	Weight gain and linear growth (g/month, cm/month)	NA
Larnkjær, 2009 (26)	Denmark	RCT	Whole milk vs. standard infant formula + fish oil vs. placebo. Breastfeeding optional.	Food records	94	9 months	3 months	Weight gain and linear growth (g/month, cm/month)	NA
Svahn, 1999 (24)	Sweden	RCT	Milk with different protein and fat contents (2.2–3.3 g/100 ml protein)	Food records	38	12 months	6 months	Weight gain and linear growth (g/day, mm/day)	NA
Tang, 2014 (27)	USA	RCT	Meat (1–2 glasses à 8 g protein) vs. cereal as complementary foods.	Food records, duplicate diets	42	6 months	3 months	BMIz, weight-for-age z-score (WAZ), weight-for-length z-score (WLZ), and length-for-age z-score (LAZ), and waist circumference (cm)	NA
Tang, 2019 (28, 29)	USA	RCT	Meat vs. dairy as complementary foods (both groups ~4 g/kg body weight [BW] protein) from 5 to 12 months of age.	Food records	53	5 months	≤19 months	Weight (kg), WAZ, WLZ, length (cm), and LAZ	NA
Beyerlein, 2017 (43)	USA, Finland, Germany, and Sweden	Prospective cohort (PC)	Total protein (percentage of energy intake [E%]) At 6 months: mean ~8.4 E%, at 12 months: ~13.8 E%, at 2 years: 15.1 E%, at 3 years: 14.7 E%, at 4 years: 14.8 E%, at 5 years: 14.6 E%.	3-day food records every 3–6 month	5,563	3 months	5 years	Weight, length, BMIz, and overweight/obesity	Sex, country, birthweight, maternal age, maternal pre-pregnancy Body Mass Index, gestational weight gain, maternal diabetes, maternal smoking in pregnancy, maternal alcohol intake in pregnancy, maternal education, and duration of breastfeeding
Braun, 2016 (39)	The Netherlands (Generation R)	PC	Total protein (mean 42.1 g; 12.9 E%) Animal protein (mean 26.5 g; 8.1 E%) Plant protein (mean 15.1 g; 4.7 E%) Amino acids	Food Frequency Questionnaire (FFQ)	3,564	1 years	8 years	Weight, height, and BMIz.	Sex, ethnicity, age, energy intake breastfeeding, playing sports, household income maternal BMI at enrolment, maternal education, folic acid use during pregnancy, smoking during pregnancy, and diet score
Durao, 2017 (44)	Portugal	PC	Total protein (mean 18.8 E% in girls (4.4 g/kg bw), 18.6 E% (4.5 g/kg bw) in boys at 4 years of age)	3-day food diaries	1,999	4 years	3 years	BMIz, weight, length, body fat, and waist circumference.	Total energy intake, maternal education, maternal BMI, birthweight, gestational age, breastfeeding, physical exercise
Garden, 2011 (37)	Australia	PC	Total protein Food groups	3-day weighed food records	362	18 months	6.5 years	BMI, overweight (BMI ≥85th %), obesity (BMI≥95th %), waist circumference	Sex, asthma study intervention group, birth weight, breastfeeding for at least 6 months, parental obesity, ethnicity, smoking in pregnancy, and father’s education, and total energy.
Gunnarsdottir, 2003 (31)	Iceland	PC	Total protein (mean ~8–9 E% at 2–6 months, 15–16 E% at 9–12 months)	48-h food records	90	2 months	6 years	BMI and overweight/obesity	Total energy and macronutrients
Günther, 2007 (34, 35)	Germany	PC	Total protein, animal protein, vegetable protein, dairy protein, meat protein, cereal protein (E%) (median total intake, 2.1 g/kg BW at 6 months, 2.7 g/kg BW at 12 months (9.8 and 13.3 E%)	3-day weighed food records	203	3–6 months	7 years	Weight, height, BMIz, overweight, and over fatness	Total energy, total fat intake, presence of siblings, maternal overweight and BMIz at baseline
Hoppe, 2004 (32)	Denmark	PC	Total protein (median 2.7 g/kg BW; ~13 E%)	5-day weighed food record	105	9 months	~9 years	Weight, height, BMI, and %BF,	Sex, parental size, and body size at 9 months.
Jen, 2019 (48)	The Netherlands (Generation R)	PC	Total protein (mean 42.1 g; 12.9 E%) Animal protein (mean 26.5 g; 8.1 E%) Plant protein (mean 15.1 g; 4.7 E%)	FFQ	3,573	1 years	9 years	Weight, height, BMIz, fat mass index (FMI), and fat-free mass index (FFMI)	Sex, ethnicity, age, total energy, birth weight SD score, breastfeeding, diet quality, screening time, playing sports, protein intake.
Rolland-Cachera, 1995 (4)	France	PC	Total protein (mean 16.3 E%)	Diet history and 24-h recall	112	2 years	6 years	BMI, skinfold thickness, and rebound of BMI	Baseline BMI and parental BMI
Morgen, 2018 (46)	Denmark	PC	Animal protein (meat and fish, dairy protein)	Interviews	36,481	6 months	6.5–10.5 years	BMI and overweight/obesity	Maternal pre-pregnancy BMI, gestational weight gain, ponderal index standardized score (SDS) at birth, BMI SDS at 5 months, BMI SDS at 12 months, SES, parity, smoking during pregnancy, and paternal BMI,
Öhlund, 2010 (36)	Sweden	PC	Total protein (mean 12.8 E%; 3.96 g/kg at 12 months of age)	5-day food records monthly from 6 to 18 months of age	127	6 months	3.5 years	Weight, height, and BMIz	Unclear
Pimpin, 2016 (42)	UK (Gemini)	PC	Total protein (median 15.8 E%)	3-day diet records	2,154	8 months	~4 years	Weight, height, BMI, and overweight/obesity	Height, total energy intake, sex, age, birth weight, and previous growth
Pimpin, 2018 (45)	UK (Gemini)	PC	Total protein Animal protein, dairy protein, milk protein, plant protein	3-day diet records	1,939	8 months	~4 years	Weight, height, BMI, and overweight/obesity	Height, total energy intake, sex, age, birth weight, previous growth, and fat intake
Scaglioni, 2000 (30)	Italy	PC	Total protein (20–22 E%)	FFQ, interviews	147	1 years	4 years	Overweight (BMI ≥ 90th %)	None
Skinner, 2004 (33)	USA	PC	Total protein (mean 14 E%)	24-h dietary recall	70	2 years	6 years	Weight, height, BMI	Unclear
Smith-Brown, 2018 (47)	Australia	PC	Protein-rich foods (animal foods, meat/fish/eggs, dairy products)	FFQ	36	12 months	1–2 years	BMIz, fat mass (FM), fat-free mass	None
Switkowski, 2019 (49)	USA	PC	Total protein (mean 58.3 g/day; 3.77 g/kg bw). Animal protein	FFQ	1,165	3.2 years (median)	4.5–10 years (median)	BMIz, skinfold thickness, FM, and LBM	Race, age, household income, maternal education, breastfeeding, maternal and paternal BMI, birth weight for GA z-score, fast food intake, and physical activity.
Thorisdottir, 2014 (38)	Iceland	PC	Total protein (mean 14.5 E%; 3 g/kg BW) Animal, dairy, meat. and fish (9.9 E%) Vegetable (4.4 E%)	3-day weighed food record	199	12 months	5 years	Weight, height, and BMI	Sex, energy intake, breastfeeding duration, maternal education, and birth weight.
Voortman, 2016 (40, 41)	The Netherlands (Generation R)	PC	Total protein (mean 42.1 g; 12.9 E%) Animal protein (mean 26.5 g; 8.1 E%) Plant protein (mean 15.1 g; 4.7 E%)	FFQ	2,965	1 year	5	Weight, height, BMIz, % body fat SDS, FMI SDS, and FFMI SDS.	Energy intake (nutrient residual method), sex, age at outcome measurement, household income, maternal age, education, BMI and smoking during pregnancy, child’s ethnicity, birth weight SDS, breastfeeding total fat intake, diet quality score, screen time and sports participation (+ BMI SDS at 1 year of age).

There were 21 publications from 16 cohort studies (4, 30–49) including between 36 (47) and 36,481 (46) (total *n* = 60,422) children for end-point assessments. One of the cohort studies included children from the United States, Finland, Germany, and Sweden (43) while the other study included children from Denmark (32, 46), Iceland (31, 38), Sweden (36), the United Kingdom (42, 45), Germany (34), the Netherlands (39–41, 48), France (4), Portugal (44), Italy (30), Australia (37, 47), and the United States (33, 49). Of all 21 publications from cohort studies, four were from the Dutch Generation R study (39–41, 48), two were from the German Dortmund Nutritional and Anthropometric Longitudinally Designed (DONALD) Study (34, 50), and two were from the Gemini study conducted in the UK (42, 45).

According to the eligibility criteria, all included studies enrolled mainly healthy, term infants or children (girls and boys), although the Gemini study on twins included a relatively high proportion born preterm (gestational age <37 weeks) (42, 45). The study with children from four countries included only those with high genetic risk for type 1 diabetes (43). In several studies, infants were recruited at birth, usually at child health’s clinics or through advertisements. Age at the start of the intervention or follow-up period ranged from 3 months to 4 years, while the age at the outcome assessment ranged from 6 months to 13 years. Children had been breastfed from birth in all but one RCT, in which only formula-fed infants were included (29). In the cohort studies, inclusion was independent of the breastfeeding status.

#### Types of intervention or exposure

Two RCTs used different dairy products or compared cow’s milk with formula (24, 26), while three RCTs compared different complementary foods consisting of meat, micronutrient-fortified cereal or dairy (25, 27, 29). In the first two trials, the intake of complementary foods was *ad libitum* in all study groups. One of the trials included fish oil vs. placebo as a co-intervention; however, this had no effect on the outcome (26). Another trial primarily assessed differences between sources of protein (dairy vs. meat-based complementary foods), so that the absolute protein intake was identical between groups (29).

In all studies, the reported intake of total protein in g/kg BW was more than adequate and within recommended E% (3), albeit not quantified in two cohort studies (46, 47). In the remaining cohorts, five studies (in seven publications) reported animal protein and plant protein separately (35, 38–40, 45, 48, 49); animal protein was further separated into dairy and non-dairy (meat/fish/egg) protein in four of those. Morgen et al. (46) only assessed protein intake from animal sources, while Smith-Brown et al. (47) only assessed servings of animal protein sources as such, not nutrients. Some cohort studies assessed dietary intake at different ages (31, 34, 35, 43).

#### Outcome assessment

The duration of the interventions in the RCTs ranged from 3 to 7 months (i.e. final outcomes were assessed at 9–18 months of age), while Tang et al. also carried out outcome assessments 1 year after the intervention at 24 months of age (29). In the cohort studies, the follow-up time between the dietary assessments and outcome ranged from 1 to 2 years to a median 10 years (i.e. from the age of 2–3 years to 13 years at follow-up).

All RCTs reported weight gain and linear growth, expressed in various units, such as g/month or g/day for weight and cm/month or mm/day for length. The RCTs by Tang et al. also reported weight-for-age z-score (WAZ), length-for-age z-score (LAZ), weight-for-length z-score (WLZ) (27, 29), and BMI z-score (BMIz) (29). None of the RCTs reported body composition indices, although one reported change in waist circumference (27). The outcomes were mostly measured by nurses or study personnel in health centers or hospitals.

Eight cohort studies (eight publications) reported associations of protein intake with BMI (31–33, 37, 38, 42, 45, 51), six (10 publications) with BMIz (34–36, 39, 40, 43, 44, 48, 49, 52), four (five publications) with fat mass index (FMI) or fat mass-index scores (FMIz) (40, 44, 47–49) and three (five publications) with percent body fat (%BF) (32, 34, 35, 40, 41), while four studies (six publications) reported associations between the total protein intake and risk of overweight and/or obesity (29, 34, 35, 42, 43, 45). Different sources of protein were assessed in four cohort studies in relation to BMIz (34, 39, 40, 47–49), in three cohorts in relation to BMI (37, 38, 45) and in two cohorts in relation to overweight and/or obesity (45, 46). Body fat indices were measured by DEXA (32, 40, 41, 48, 49) or bioimpedance (44), or calculated from skinfold thicknesses (34, 35) or total body water (47). Öhlund et al. did assess body fat but not its relation with protein intake (36). Overweight and obesity were classified according to the International Obesity Task Force cutoffs (34, 35, 42, 45, 46) as BMIz >1 or >2 (43), and BMI >75th (34) or ≥90th percentiles (30).

### Risk of bias in included studies

**Fig. 2:**
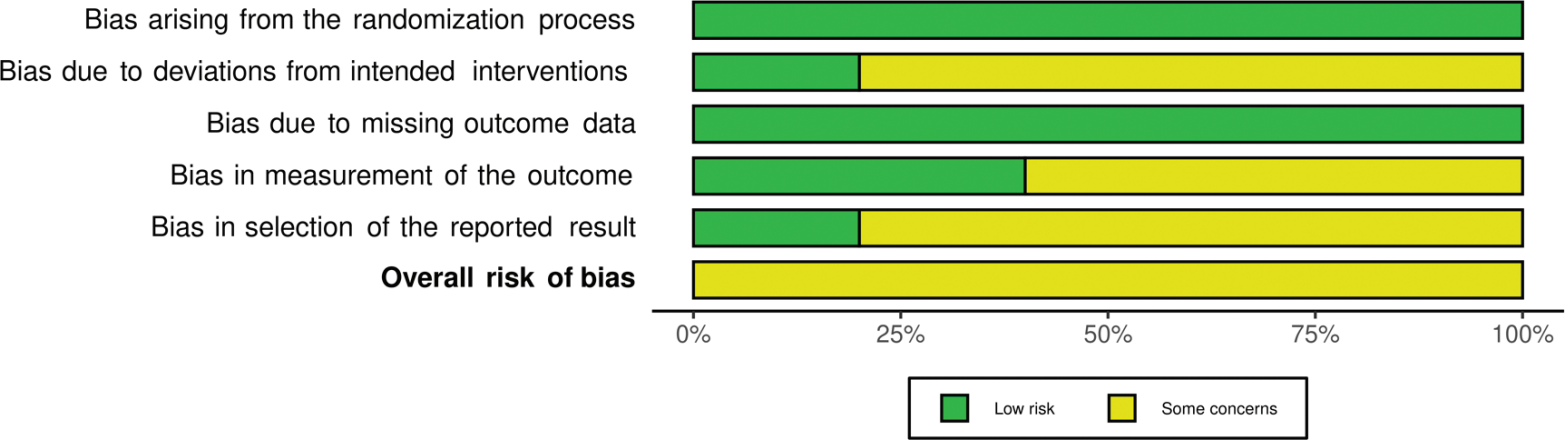
Summary risk of bias per domain in randomized controlled trials.

The risk of bias assessment per domain in RCTs is outlined in Fig. 2 and Supplementary Fig. S1. All RCTs had overall some concerns for risk of bias, mostly related to potential awareness of the assigned interventions among the caretakers and outcome assessors, and the lack of reported pre-specified analysis plans (with two exceptions [26, 29]), increasing the risk of reporting selected results. However, outcomes had objective measurements and would therefore likely not be influenced by detection bias. For the domains of randomization, deviations from the intended interventions, and missing data, all trials were deemed to be low for risk of bias; however, the method of random sequence generation was unclear in all but two RCTs (26, 28, 29).

**Fig. 3:**
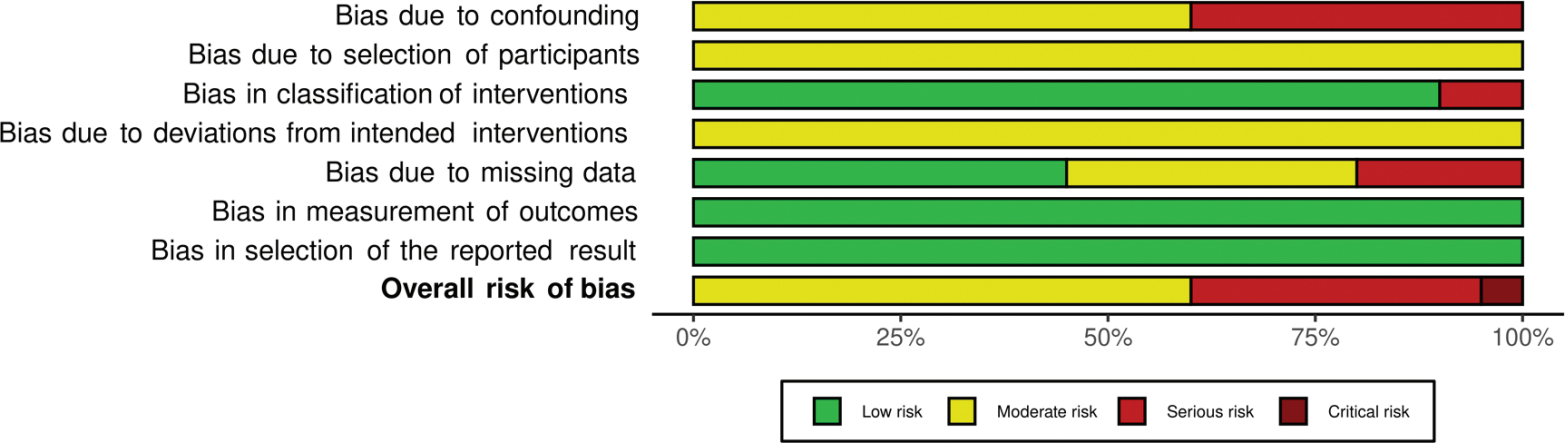
Summary risk of bias per domain in prospective cohort studies.

Prospective cohort studies were mostly judged as moderate in overall risk of bias. Due to the potential for confounding inherent in all observational studies, none of the cohort studies reported a low risk of bias for all domains; however, most had a moderate risk of bias in exposure classification, outcome measurement, and the reporting of results (Fig. 3 and Supplementary Fig. S2).

### Results per outcome

#### Body weight, length/height, and BMI

*Randomized controlled trials*. Due to heterogeneity in types of intervention and reported outcomes, meta-analysis was not performed with the RCTs; instead, the individual study findings are summarized in Table 4. All RCTs (*n* = 5) found generally null effects of either high-protein milk (24, 26) or complementary foods (25, 27, 29) on weight gain, but also indicating that lower protein diets did not negatively affect growth in these infants.

**Table 4:** Summary of findings in randomized controlled trials^1^

Author (alphabetical order), year	Outcomes reported	High-protein intervention	Lower protein intervention(s)/control
Krebs, 2006	Weight gain and length gain	*N* = 46 (4–7 months) / 30 (7–12 months) Meat: Weight gain^2^: 4–7 months: Δ399 (±136) g/month 7–12 months: Δ267 (±93.5) g/month Length gain^2^: 4–7 months: Δ1.62 (±0.42) cm/month 7–12 months: Δ1.25 (±0.25) cm/month	*N* = 42 (4–7 months) / 26 (7–12 months) Cereal: Weight gain^2^: 4–7 months: Δ427 (±136.1) g/month 7–12 months: Δ252 (±91.8) g/month Length gain^2^: 4–7 months: Δ1.78 (±0.42) cm/month 7–12 months: Δ1.27 (±0.24) cm/month
Larnkjær, 2009	Weight gain and length gain	*N* = 38 Whole milk (~3.4 g/100 mL protein): Weight gain: Δ257 (±103) g/month Length gain: Δ1.25 (±0.34) cm/month	*N* = 45 Standard infant formula (≤1.5 g/100 mL protein): Weight gain: Δ242 (±111) g/month Length gain: Δ1.24 (±0.28) cm/month
Svahn, 1999	Weight gain and length gain	*N* = 17 Cow’s milk^3^: Weight: Δ7.8 g/day (range 2.8–12 g/day) Length: Δ0.4 mm/day (range 0.26–0.62 mm/day)	*N* = 9 Protein-reduced milk: Weight: Δ7.9 g/day (range 4.7–11.5 g/day) Length: Δ0.4 mm/day (range 0.28–0.51)
Tang, 2014	weight-for-age z-score (WAz) length-for-age z-score (LAz) weight-for-length z-score (WLz) BMIz Waist circumference (WC)	*N* = 14 Meat: WAZ: Δ0.32^4^ LAZ: Δ0.08 WLZ: Δ0.29 BMIz: Δ0.39 WC: Δ2 cm	*N* = 28 Cereal: WAZ: Δ -0.14^4^ LAZ: Δ0.45 WLZ: Δ0.08 BMIz: Δ0.21 WC: Δ2 cm
Tang, 2019	Weight, length WLz (in figure) LAz (in figure) Waz (in figure)	*N* = 26 Dairy: Weight at 24 months: 12.4 (1.5) kg Length at 24 months: 87.1 (3.3) cm WLz: n.d. Waz: n.d. LAz: -0.37 (±0.88)	*N* = 27 Meat: Weight at 24 months: 12.6 (1.0) kg Length at 24 months: 89 (2.3) cm WLz: n.d. Waz: n.d. LAz: 0.19 (±0.52)

1 BMI: Body Mass Index. LAZ: length-for-age z-score. N.d.: no data. WAZ: weight-for-age z-score. WLZ: weight-for-length z-score. WC: waist circumference.

2 Standard deviations calculated from SE (SE x √n).

3 Cow’s milk = mean of low-fat + standard-fat milk groups.

4 Change calculated as difference in mean at 9 vs. 5 months of age (from the Supplementary data).

The RCTs assessing meat as complementary foods did not report significant effects on weight, length, or WLZs compared with low-protein cereal (25, 27). Tang et al. (29) found an increase in LAZ in the meat group compared with the dairy group from 5 to 12 months. This difference persisted after the end of the intervention (12 months) period up to 24 months of age (mean difference in length 1.9 cm). Weight for age was not significantly different at the 24-month follow-up period.

None of the RCTs reported results of fat mass; however, only Tang et al. (27) reported change in waist circumference.

#### Prospective cohort studies

*Total protein and weight/BMI*. Among the 13 prospective cohort studies assessing the total protein intake and weight and/or BMI/BMIz, eight (10 publications) reported a significant positive or direct association (Table 5) (4, 32, 33, 36–40, 42, 48). The remaining five studies showed positive associations that were limited to one sex or specific age groups; three studies found positive associations in boys, but not in girls (31, 44, 49), and one of the studies reported a positive association between protein intake at 4–5 years of age, but not earlier, and BMIz at 5.5 years (43). Finally, one cohort found significant associations with the outcomes at 7 years of age when looking at total protein intake at 1 and 5–6 years of age, but not at 6 months, 1.5–2 or 3–4 years of age, and a significant, positive association with a consistently high intake from 12 to 18–24 months compared with a consistently low intake at these ages (34, 35). None of the studies found an inverse association between protein intake and weight and/or BMI/BMIz.

**Table 5:** Summary of findings from cohort studies – total protein intake^1^

Author, year	Age at outcome (y) (ordered by age)	Outcomes reported (in final models)	Findings (from final models)^2^	Effect size	RoB
Öhlund, 2010 (36)	4	BMIz	↑ BMIz	Per g/day protein: - BMI +0.042 SDS	Serious
Pimpin, 2016 (42)	5	Weight, height, body mass index (BMI), and overweight/obesity	↑ Weight, ↑ BMI, ↔ height, ↔ overweight/obesity	At 5 years of age: Per 1 percentage of energy intake (E%) protein: - BMI 0.043 kg/m^2^ - Weight 0.052 cm - Overweight and/or obesity, OR 0.93 (95%CI 0.81, 1.07)	Moderate
Scaglioni, 2000 (30)	5	Overweight (BMI >90th %)	↑ overweight	Protein intake: Overweight at 5 years: 22 E%, Not overweight at 5 years: 20 E%. Positive association with overweight (*P* = 0.05, OR not reported)	Serious
Beyerlein, 2017 (43)	5.5	Overweight/obesity (BMIz >1/>2)	Intake after 3.5 and 4.5 years of age: ↑ Overweight/obesity. Intake at earlier ages: not significant (ns)	Per 1 E% protein: - Overweight: OR = 1.03 (95% CI 1.02, 1.05) - Obesity: OR = 1.12 (1.08, 1.16)	Moderate
Voortman, 2016 (40, 41) (Generation R)	6	Weight, height, BMIz, % body fat standardized score (SDS), fat mass index (FMI) SDS, fat-free mass index (FFMI) SDS.	↑ BMI SDS, ↑ FMI SDS, ↑ body fat percentage (%BF), ↔ FFMI SDS	Per 10 g/day protein: - BMIz +0.05 - FMI SDS +0.06 - %BF +0.06 SDS	Moderate
Gunnarsdottir, 2003 (31)	6	BMI	Boys: ↑ BMI Girls: ns All: ns	Per E% protein: Boys, intake at 9 and 12 months: - BMI +0.2 kg/m^2^. Boys, intake at 6 months: ns. Boys, intake at 9 and 12 months: - Q4 vs. Q1: +2.5 kg/m^2^. BMI Girls: ns All: ns	Serious
Thorisdottir, 2014 (38)	6	Weight, height, and BMI	↑ BMI, ↔ weight, ↔ height	Per 1 E% protein: - BMI +0.08 kg/m^2^. Q4 vs. Q1: - BMI +0.8 kg/m^2^. - Weight +1.2 kg (ns) - Height +0.1 cm (ns)	Moderate
Durao, 2017 (44)	7	BMIz, body fat, waist/height	Boys: ↑ BMIz, ↑ FMI, ↑ waist-to-height ratio ratio^3^ Girls: ns	Per g/day protein: Boys: - BMIz +0.205 Girls: - BMIz +0.11 (ns)	Moderate
Günther, 2007 (34, 35)	7	BMIz, overweight, and over fatness	Intake at 12 months: ↑ BMI SDS, ↑ %BF Intake at 6 months, 1 year, 1.5–2 years and 3–4 years: ns High intake at both 12 and 18–24 months: ↑ BMI SDS, ↑ %BF, ↑ risk of overweight, ↑ risk of over fatness	Per 1 E% protein: Intake at 12 months: - BMI SDS ~ +0.2^4^ - ln %BF ~ +0.04^2^ High intake at 12 and 18–24 months vs. low intake: - Risk of overweight OR = 2.39 (95% CI: 1.14, 4.99) - Risk of over fatness OR = 2.28 (95% CI: 1.06, 4.88)	Moderate
Garden, 2011 (37)	8	BMI and waist circumference	↑ BMI, ↔ WC	Per 1 E% protein: - BMI +0.12 kg/m^2^ - WC +0.24 cm (ns)	Moderate
Rolland-Cachera, 1995 (4)	8	BMI and skinfold thickness	↑ BMI↑ subscapular skinfold thickness (total body fat) ↔ triceps skinfold thickness (%BF)	Correlation coefficients: - BMI, *r* = 0.22 - Subscapular skinfold, *r* = 0.20 - Triceps skinfold, *r* = 0.11 (ns)	Critical
Skinner, 2004 (33)	8	BMI	↑ BMI	Per g/day protein: - BMI +0.01 kg/m^2^	Serious
Braun, 2016 (39) (Generation R)	Up to 9	Weight, height, and BMI SDS	↑ Weight SDS,↑ height SDS, ↑ BMI SDS	Per 10 g/day protein: - Weight +0.06 SDS - Height +0.03 SDS - BMI +0.05 SDS	Moderate
Jen, 2019 (48) (Generation R)	Up to 10	Weight, height, BMIz, FMI, and FFMI	↑ Weight SDS, ↔ Height SDS,↑ BMI SDS, ↑ FMI SDS, ↔ FFMI	Per 5 E% protein: - Weight +0.11 SDS - Height +0.05 SDS - BMI +0.11 SDS - FMI +0.09 SDS - FFMI +0.03 SDS	Moderate
Hoppe, 2004 (32)	10	Weight, height, BMI, and %BF	↑ Weight, ↔ BMI, ↔ %BF	Per 1 E% protein: - BMI +0.097 kg/m^2^ (ns) - Weight +0.44 kg - Height + 0.51 cm - %BF +0.13 (ns)	Serious
Switkowski, 2019 (49)	7.7 and 13	BMIz, skinfold thickness, FMI, lean body mass index	At 13 years of age: Boys: ↑ BMIz, ↔ skinfolds, ↔ lean mass index, ↔ FMI Girls: ns	Per 10 g/day protein Boys: - BMI +0.12 SDS	Moderate

1 BMI: Body Mass Index. BW: body weight. E%: percentage of energy intake. FFM: Fat-free Mass. FFMI: Fat-free Mass Index. FM: Fat Mass. FMI: Fat Mass Index. LBMI: Lean Body Mass Index. ns: not significant. SDS: standardized score. W/ht ratio: waist-to-height ratio. z: z-score. %BF: body fat percentage.

2 Arrows indicate the direction of the association.

3 FMI and W/ht ratio significant with both high-protein intake and high GL.

4 Estimated from the figure.

**Fig. 4:**
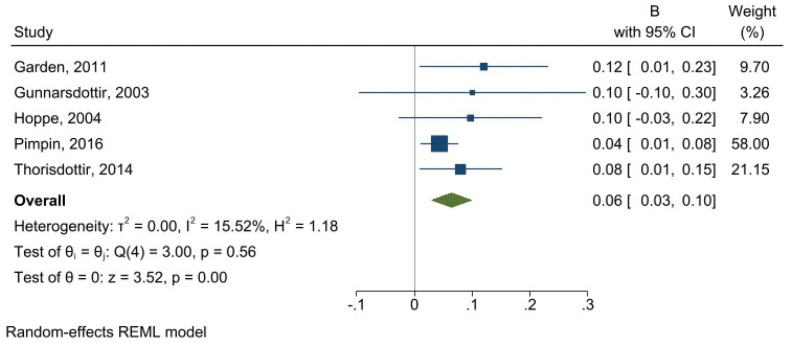
Forest plot showing associations between 1 E% increment in total protein intake and body mass index (BMI). Note: For Gunnarsdottir et al., 95% confidence intervals were calculated from reported standard errors. REML = restricted maximum likelihood.

The requirement for a meta-analysis was fulfilled by five prospective cohort studies assessing linear associations between E% of total protein in relation to BMI (*n* = 2,458 with intake and BMI measurements available) (31, 32, 37, 38, 42), with additional data provided by the study authors of one study (31). Subgroup analysis and formal publication bias tests were not feasible due to the few studies. We observed an overall pooled effect estimate of 0.06 BMI kg/m^2^ (95% CI 0.03, 0.10) per one E% increment in total protein (Fig. 4) with overall low heterogeneity (*I*^2^ =15.5). Among these, Gunnarsdottir et al. found a significant, positive association only in boys, not in girls nor the overall sample (31).

The five included studies in the meta-analysis applied regression models differing in number and types of covariates. Total energy intake was not adjusted for in one study (32). One study did not adjust for sex (31). Three adjusted for birth weight (37, 38, 42), while two adjusted for breastfeeding (37, 38) and for parental weight status (32, 37). Other dietary variables were not included in any model. The studies had either moderate (37, 38, 42) or serious (31, 32) overall risk of bias. A few other studies instead reported the total protein intake in g/day (1, 27), or by comparing extreme quartiles or quintiles (1, 25, 36, 44). All these studies reported associations with significantly higher BMI.

Studies that assessed BMIz instead of absolute BMI found increases by 0.04–0.16 SDS per gram/day of total protein (36, 44), 0.05–0.12 (in boys) SDS per 10 g/day protein (39, 40, 49), 0.2 SDS per 1 E% protein (34), and 0.11 SDS per 5 E% protein (48).

#### Animal vs. vegetable protein and weight/BMI

Differential associations with animal and plant protein for BMI/BMIz were reported in eight publications (35, 37–40, 45, 48, 49) – two studies assessed sources of animal protein alone (46, 47). Garden et al. also assessed associations with food groups, including dairy and meats, but not protein intake from these as such (37) (Table 6).

**Table 6:** Summary of findings from cohort studies – animal or plant protein sources^1^

Author, year	Age at outcome (y) (ordered by age)	Outcomes	Findings^2^	Effect sizes	RoB
Smith-Brown, 2018 (47)	2–3	BMIz, FM, FFM, W/ht ratio	Animal protein food: ↔BMIz, ↑ FFMIz, ↔, FMI, ↔ W/htz Dairy: ↔BMIz, ↑ FFMIz, ↔, FMI, ↔ W/htz Meat, fish, eggs: ↔ BMIz, ↑ FFMIz, ↔ FMI, ↑ W/htz	Correlation coefficients: Animal protein foods: - BMIz, *r* =0.35 (ns) - FFMIz, *r* = 0.58 - FMIz, *r* = -0.221 (ns) - W/htz, *r* = 0.37 (ns) Dairy: - BMIz, *r* = 0.29 (ns) - FFMIz, *r* = 0.51 - FMIz, *r* = -0.24 (ns) - W/htz, *r* = 0.26 (ns) Meat, fish, and eggs: - BMIz, *r* = 0.39 (ns) - FFMIz, *r* = 0.53 - FMIz, *r* = -0.084 (ns) - W/htz, *r* = 0.55	Serious
Pimpin, 2018 (45)	5	Weight, BMI, and overweight/obesity	Animal protein (non-dairy): ↔ weight, ↔ BMI, ↔ overweight/obesity Dairy protein: ↑ weight, ↑BMI, ↔ overweight/obesity^3^ Milk protein: ↑ weight, ↑ BMI, ↑ overweight/obesity Plant protein: ↔ weight, ↔ BMI, ↔ overweight/obesity Low dairy-high plant protein diet: ¯ weight, ↔ BMI, ↔ overweight/obesity	Per 1 E% protein: Animal protein: - Weight +0.02 kg (ns) - BMI +0.004 kg/m^2^ (ns) - Overweight/obesity OR = 0.99 (95% CI: 0.91, 1.09). Dairy protein: - Weight +0.046 kg - BMI +0.037 kg/m^2^ - Overweight/obesity OR = 1.07 (0.98, 1.16)^3^ Milk protein: - Weight +0.046 kg - BMI +0.065 kg/m2 - Overweight/obesity OR = 1.12 (1.04, 1.22) Plant protein: - Weight +0.005 kg (ns) - BMI +0.024 (ns) - Overweight/obesity OR = 0.93 (0.8, 1.09). Low dairy-high plant protein diet: - Weight -0.05 kg - BMI -0.065 kg/m2 (ns) - Overweight/obesity OR = 0.84 (0.70, 1.00)	Moderate
Voortman, 2016 (40, 41)	6	Weight, height, BMI SDS, FMI SDS, and FFMI SDS.	Animal protein: ↑ BMI SDS, ↑ FMI SDS, ↔ FFMI SDS Vegetable protein: NS	Per 10 g/day protein: - Animal protein: - BMI +0.06 SDS - FMI +0.05 SDS - FFMI +0.02 SDS (ns) Vegetable protein: - BMI +0.01 SDS (ns) - FMI -0.01 SDS (ns) - FFMI 0.00 (ns)	Moderate
Thorisdottir, 2014 (38)	6	Weight, height, and BMI	Animal protein: ↑ BMI, ↑ weight, ↔ height (dairy or meat/fish protein: ns) Vegetable protein: ↔ BMI, ↔ weight, ↔ height	Per E% protein: - Animal protein: BMI 0.07 kg/m^2^. - Vegetable protein: BMI -0.08 kg/m^2^ (ns) Q4 vs. Q1: - Animal protein: weight +1.5 kg, height +0.9 cm (ns). - Vegetable protein: weight -0.8 kg (ns), height +0.2 cm (ns)	Moderate
Günther, 2007 (35)	7	BMIz and body fat	Intake at 12 months: Animal protein: ↑ BMI SDS, %BF Vegetable protein: ↔ BMI SDS, ↔ %BF Dairy protein: ↑ BMI SDS, ↔ %BF Meat protein: ↔ BMI SDS, ↔ %BF Cereal protein: ↔ BMI SDS, ↔ %BF Intake at 5–6 years: Animal protein: ↑ BMI SDS, ↑ %BF Vegetable protein: ↔ BMI SDS, ¯ %BF Dairy protein: ↔ BMI SDS, ↔ %BF Meat protein: ↔ BMI SDS, ↔ %BF Cereal protein: ↔ BMI SDS, ↔ %BF	3rd vs. 1st tertile of E% protein: Animal protein - 12 months: ◦ BMI +0.43 SDS ◦ % BF +2.01 - 5–6 years: ◦ BMI +0.12 SDS (ns) ◦ %BF +1.19 Vegetable protein: - 12 months ◦ BMI +0.05 SDS (ns) ◦ %BF -0.36 (ns) - 5–6 years ◦ BMI -0.13 SDS (ns) ◦ %BF -0.53 Dairy protein: - 12 months ◦ BMI +0.32 SDS ◦ %BF +1.11 (ns) - 5–6 years ◦ BMI +0.10 SDS (ns) ◦ %BF +0.8 (ns) Meat protein: - 12 months ◦ BMI -0.08 SDS (ns) ◦ % BF -1.36 (ns) - 5–6 years: ◦ BMI +0.10 SDS (ns) ◦ %BF +0.37 (ns) Cereal protein: - 12 months ◦ BMI +0.14 SDS (ns) ◦ %BF +0.8 (ns) - 5–6 years ◦ BMI -0.04 SDS (ns) ◦ %BF -0.3 (ns)	Moderate
Garden, 2011 (37)	8	BMI and waist	Dairy foods: ↔BMI, ↔ WC Milk: ↔BMI, ↔ WC Meats: BMI, WC	Per quintile of g/day protein: Dairy foods: - BMI -0.2 kg/m^2^ (ns) - WC -0.51 cm (ns) Milk: - BMI -0.11 kg/m^2^ (ns) - WC -0.30 cm (ns) Meats: - BMI +0.25 kg/m^2^ - WC +0.59 cm	Moderate
Braun, 2016 (39)	Up to 9	Weight, height, and BMI SDS	Animal: weight SDS, height SDS, BMI SDS Dairy and non-dairy protein: Weight SDS, BMI SDS, ↔ Height SDS Vegetable: ns	Per 10 g/day protein: Animal protein: - Weight +0.07 SDS - Height +0.04 SDS - BMI +0.06 SDS Vegetable protein: - Weight +0.01(ns) - Height +0.01 (ns) - BMI +0.01 (ns)	Moderate
Jen, 2019 (48)	Up to 10	Weight, height, BMIz, FMI, and FFMI	Animal protein: weight SDS, height SDS, BMI SDS, FMI SDS, ↔ FFMI SDS Vegetable protein: NS	Per 5 E% protein: Animal protein: - Weight +0.12 SDS - Height +0.06 SDS - BMI +0.11 SDS - FMI +0.09 SDS - FFMI +0.03 SDS (ns) Dairy protein: - Weight +0.13 SDS - Height +0.07 SDS (ns) - BMI +0.12 SDS - FMI +0.06 SDS (ns) - FFMI +0.01 SDS (ns) Non-dairy animal protein: - Weight +0.10 SDS - Height +0.05 SDS (ns) - BMI +0.11 SDS - FMI +0.13 SDS - FFMI +0.06 SDS (ns) Vegetable protein: - Weight +0.05 (ns) - Height +0.03 (ns) - BMI +0.04 SDS (ns) - FMI -0.02 SDS (ns) - FFMI +0.07 SDS (ns)	Moderate
Morgen, 2018 (46)	7 and 11	BMI and overweight/obesity	At 11 years of age^4^: Dairy protein: ↔BMIz, ↔ overweight Meat/fish protein: BMIz, ↔ overweight	At 11 years of age: Dairy (per 5 g/day): - BMIz -0.003 (ns) - Overweight: OR = 0.96 (95% CI: 0.89, 1.04) Meat and fish (per 2 g/day): - BMIz +0.013 - Overweight: OR = 1.01 (0.95, 1.07)	Serious
Switkowski, 2019 (49)	7.7 and 13	BMIz, height, and LBM	Animal protein: BMIz, ↔ height, LBMI (in boys only), ↔ FMI Plant protein: ns	NR	Moderate

1 BMI: body mass index. BW: body weight. E%: percentage of energy intake. FFM: fat-free mass. FFMI: Fat-free Mass Index. FM: fat mass. FMI: Fat Mass Index. FFQ: food frequency questionnaire. LBMI: Lean Body Mass Index. ns: not significant. RoB: risk of bias. SDS: standardized score. W/ht: waist-to-height ratio. z: z-score. %BF: body fat percentage.

2 Arrows indicate the direction of association.

3 Significantly increased OR for overweight/obesity at 3 years of age, but not 5 years.

4 ↑ BMIz only at 7 years of age for dairy. ↑ BMIz, ↑ overweight only at 7 years of age for meat and fish.

A higher intake of total animal protein, compared with lower, was associated with higher weight and BMI/BMIz in four out of eight cohorts (35, 38–40, 48, 49), although only in boys in the study by Switkowski et al. (49), while none of the studies found such associations for plant protein. Within different sources of animal protein, dairy protein was significantly associated with increased BMI/BMIz in four studies (35, 39, 45, 46, 48); however, Günther et al. (35) only found a significant association for dairy protein intake at 12 months, not 5–6 years of age, and Morgen et al. (46) only found a significant association with BMIz at 7, not 11, years of age. Non-dairy animal protein (e.g. meat protein) was positively associated with BMIz in two of these cohorts (39, 46, 48). Dairy foods or milk as such (i.e. not specifically dairy protein) was not associated with BMIz in two studies (37, 47), while meat was positively associated with BMI in one study (37). The positive associations between animal protein and BMI/BMIz reflected the associations found with total protein in the same cohorts.

#### Total protein and body composition

Associations between total protein intake and body fat percentage (%BF) (32, 34, 40), fat mass and/or or fat-free mass (40, 44, 48, 49) were reported in six publications (five cohorts). Four papers also reported waist circumference or waist–height ratios (37, 40, 41, 44).

One of the studies found no significant association between total protein intake and %BF (32); one found a positive association between protein intake and %BF, with a stronger association in girls than boys (40, 41), while Günther et al. found a positive association between total protein intake at 12 months, but not earlier, and %BF at 7 years of age (34, 35). In addition, Rolland-Cachera used skinfold thickness as a proxy for body fat; protein intake was significantly associated with subscapular (indicating total body fat), but not with triceps (indicating %BF) skinfold thicknesses (4). However, Switkowski et al. found no association with skinfold thickness (subscapular and triceps combined) (49).

There were limited findings regarding fat mass/fat-free mass, that is, positive associations with FMI and null associations with the fat-free mass index in the Generation R cohort (40, 48) but null associations with both fat mass and lean mass in the study by Switkowski et al. (49). In the two studies reporting waist circumference or waist–height ratio, no significant associations with total protein intake were found (37, 44).

#### Animal vs. vegetable protein and body composition

Only two studies reported on animal protein intake and fat mass or fat-free mass with inconsistent results (40, 48, 49). Jen et al. (48) only found a significant association for total animal and non-dairy animal protein. Günther et al. also found a significant association between total animal protein intake and %BF, but not for specific animal protein sources (35). Dairy foods or milk was not associated with waist circumference or waist–height ratio, but meats/fish/eggs was, in two studies (37, 47). Smith-Brown et al. also found a positive association between animal protein foods and fat-free mass (47).

#### Total protein intake and risk of overweight and/or obesity

Few studies assessed the relationships between protein intake and odds of overweight and/or obesity, and the findings were mainly in the direction of increased odds. Beyerlein et al. found increased risk of overweight and obesity in 5.5-year-old children with higher protein intake per 1 E% increment at 3.5 and 4.5 years of age, that is, OR 1.03 (95% CI 1.0, 1.05) for overweight and OR 1.12 (1.08, 1.16) for obesity (43), while Pimpin et al., assessing protein intake at 8 months of age, did not (42). Günther et al. found a consistently high-protein intake from 12 to 18–24 months compared with lower intake associated with increased risk of overweight, defined as >75th percentile of the German reference curves: OR 2.39; 95% CI 1.14, 4.99 (34), while Scaglioni et al. found a higher baseline intake of protein (E%) in children at 1 year of age in those with overweight (BMI >90th percentile) than without overweight at 5 years of age (30).

#### Animal vs. vegetable protein intake and risk of overweight and/or obesity

Only two of studies assessing sources of protein reported risk of overweight and/or obesity. In contrast with their null findings for total protein, Pimpin et al. found a higher risk of overweight and/or obesity at 5 years of age with higher intake of fluid milk protein, but not other animal or dairy protein sources or plant protein (45). There was a borderline inverse association (OR 0.84; 95% CI 0.70, 1.00) between the intake of a low dairy/high plant protein dietary pattern and risk of overweight and/or obesity. Morgen et al. (46) only assessed animal protein sources and found no significant associations between dairy or meat/fish protein and overweight at 11 years of age. However, meat and/or fish protein was positively associated with the risk of overweight at 7 years of age.

### Certainty in the evidence

As described above and in Table 4, only one RCT evaluated protein intake on BMI/BMIz (27), finding no effect, but as this study did not directly test the effects of protein intake per se, was small and had a short intervention period, it does not provide strong evidence against the effect of protein. The association between total protein and BMI/BMIz was assessed in 12 independent cohorts, of which 11 found positive associations in the total samples or in subgroups, further corroborated in the meta-analysis of five cohort studies, showing a positive, dose–response increase in BMI per E% higher total protein intake, with little inconsistency regarding the directions. In summary, the evidence of a positive relationship between total protein intake and BMI was considered *probable* (Table 7). Furthermore, there was *probable* evidence for an association between higher intake of animal protein and increased BMI, while the evidence was considered *probable* for no effect of plant protein on BMI.

**Table 7:** Summary of outcomes and strength of evidence

Outcome	Exposure or intervention	Number of participants^1^ (number of independent studies)	Effect (direction and number of studies)	Strength of evidence
Body mass index/BMIz	Total protein	Cohorts: 9,462 (12) Randomized controlled trials (RCTs): 42 (1)	Cohorts:↑12 RCTs: ↔	Probable
	Animal protein	Cohorts: 29 083 (8) RCTs: 42 (1)	Cohorts:↑7^2^ RCTs: ↔	Probable
	Plant protein	Cohorts: 6,645 (5) RCTs: 0	Cohorts: ↔	Probable (no effect)
Weight gain/weight for age	Total protein	Cohorts: 5,860 (4) RCTs: 292 (5)	Cohorts: 3 RCTs:↑1	Limited (suggestive)
	Animal protein	Cohorts: 5,760 (3) RCTs: 207 (4)	Cohorts:↑3^3^ RCTs:↑1	Limited (suggestive)
	Plant protein	Cohorts: 5,760 (3) RCTs: 0	Cohorts: ↔	Probable (no effect)
Length gain/length for age	Total protein	Cohorts: 4,695 (4)	Cohorts: ↔ RCTs: ↔	Limited (inconclusive)
	Animal protein	Cohorts: 4,673 (3)	Cohorts:↑1 RCTs:↑1	Limited (inconclusive)
	Plant protein	Cohorts: 4,673 (3)	Cohorts: ↔	Limited (inconclusive)
Body fat percentage/Fat Mass Index	Total protein	Cohorts: 6,368 (6)	Cohorts:↑3	Limited (suggestive)
	Animal protein	Cohorts: 4,303 (4)	Cohorts:↔3	Limited (inconclusive)
	Plant protein	Cohorts: 4,278 (3)	Cohorts: ↔	Limited (suggestive)
Overweight/obesity	Total protein	Cohorts: 6,798 (4)	Cohorts:↑3	Limited (suggestive)
	Animal protein	Cohorts: 10,105 (2)	Cohorts:↑1^4^	Limited (inconclusive)
	Plant protein	Cohorts: 1,534 (1)	Cohorts: ↔	Limited (inconclusive)

1 N participants with the respective outcomes and exposure.

2 Of which only meat (no dairy) in two studies and only dairy in one study.

3 Only dairy in one study.

4 Only milk.

One of the RCTs found a significant effect of protein intake (source) on weight gain (27), that is, a positive association between protein and weight for age, but not on weight for length or BMI. All RCTs were small, had a short-term intervention, and with varying differences in amounts of protein in the ‘high’ vs. ‘low’ groups. Of the cohorts reporting associations between total protein and weight, three of four found a significant, positive association. Due to the limited number of high-quality studies and somewhat inconsistent results, the evidence for a causal effect of total protein and/or animal protein on excessive weight gain was considered *limited but suggestive.* There was *probable* evidence for no effect of plant protein on excessive weight gain.

Regarding body composition, no RCT assessed effects on body fat or fat-free mass. Of the six cohorts assessing associations between total protein or animal protein and body fat (including skinfold thicknesses), both positive and no significant associations were found. The evidence for a positive effect of total protein on body fat was considered *limited but suggestive*, while the evidence for animal protein was *limited and inconclusive* due to few studies and inconsistent conclusions. However, the results of plant protein intake and body fat were consistent (i.e. no association), albeit with few studies. Evidence for a lack of association was therefore considered *limited but suggestive*.

There was also *limited, suggestive* evidence for an effect of total protein intake and higher risk of overweight and/or obesity, as positive associations were found in three of four cohorts; however, the results were difficult to compare directly due to methodological heterogeneity. Conclusions could not be made on the associations between animal or plant protein intake and the risk of overweight or obesity, that is, the evidence was considered *limited* and *inconclusive*.

## Discussion

This systematic review found some support for an association between higher intake of protein and increased BMI or BMI z-scores in healthy, well-nourished children based on prospective cohort studies alone, including a *de novo* meta-analysis of five prospective studies. There is also some evidence that this association is driven by protein from animal sources, while the intake of protein from plant sources was not associated with later BMI or body fat. Available data on protein intake and subsequent risk of overweight or obesity are limited, and the findings are unclear, possibly due to limited power.

The seemingly discordant results between RCTs and prospective studies in this systematic review, where the RCTs, in contrast with the cohort studies, generally observed null effects, need to be carefully appraised. Although inference with respect to causality is usually in favor of RCTs, several methodological constraints, such as short intervention periods, small sample sizes, and the nature of the interventions and comparisons, clearly affect the possibility to obtain significant findings in these studies. In this context, it is important to note based on few studies that no adverse effects on growth from protein-reduced diets compared with high-protein diets were observed in the RCTs. In the light of the need for the population within the Nordic countries and elsewhere, to move to a more plant-based diet, more probative studies assessing the safety and adequacy of lower protein diets for young children are needed.

### Comparison with other reviews

A systematic review by Hörnell et al., for the NNRs 2012, assessed protein intake in relation to growth and development, in children up to 18 years of age from studies published in 2000–2012 (5). Based on 13 studies, including one RCT (53), the authors concluded the evidence to be *convincing* that ‘higher protein intake in infancy and early childhood is associated with increased growth and higher BMI in childhood’, while there was *limited-suggestive* evidence for a stronger association with growth of animal protein, especially from dairy, compared with vegetable protein. Hörnell et al. included a wider age range and also infant formula trials such as the large European Childhood Obesity Project RCT (53).

More recently, Stokes et al. reviewed the association between protein intake in infants and children up to 2 years of age and childhood obesity-related outcomes in a systematic review including only prospective cohort studies (8). Several studies included in this review were also included by Stokes et al. (30–32, 34, 35, 38–42, 45, 46, 48), and they performed a meta-analysis on total protein intake and BMI with three of the five studies included in our meta-analysis, obtaining similar results. In another recent systematic review, Ferré et al. looked at associations between protein intake specifically during the second year of life and weight gain and overweight and/or obesity in childhood (7). They did not include any of the RCTs, but several of the same cohort studies as in the present systematic review, and concluded that there are ‘indications’ for an effect of protein intake during the second year of life and obesity risk. One of the RCTs included by Ferré et al. found that infants randomized to a protein-reduced milk from 12 months of age had reduced FMI and %BF at 2 years of age compared with those on standard cow’s milk consumption (54). However, the intervention milk was also fortified with micronutrients and synbiotics, and thus could not be included in our systematic review.

Other recent systematic reviews have focused on the effects of protein intake specifically from infant formulas and growth and weight-related outcomes in RCTs (55, 56), thus finding that lower protein formulas seem to lead to adequate growth in the short term. They included studies mostly in infants younger than our eligibility criteria.

Effects of complementary foods and beverages, rather than protein per se, have also been reviewed, including in a 2019 systematic review prepared for the USDA (57). Although the research questions were not directly comparable, they found moderate evidence that the amount of meat as complementary foods did not favorably or unfavorably affect growth or body composition, while there was insufficient evidence for effects on overweight and/or obesity.

### Potential mechanisms

Several hypotheses have been suggested to explain the link between higher intake of protein – driven by animal protein – and increased BMI in healthy, well-nourished children. One of them concerns the effects of amino acids on insulin and insulin-like growth factor 1 (IGF-I), an anabolic factor that promotes growth and adipogenesis (58). Especially branched-chained amino acids (BCAA) are hypothesized to stimulate insulin/IGF-1 secretion, and a higher intake of BCAA has also been associated with overweight in children (59). We did not include potentially mediating variables such as insulin or IGF-1 in this systematic review, and can therefore not allude to the mechanisms behind the observed associations.

We did not systematically assess the evidence for certain critical time periods for ‘programming’ of adiposity (58); however, the included studies did not show any clear pattern. For instance, the DONALD study found that a high-protein intake at 12 months of age and later, but not at 6 months, was associated with later overweight, suggesting, according to the authors, that protein intake during the period of complementary feeding and the transition to the family’s diet was decisive (34). However, positive associations between protein intake and BMI were also reported in studies assessing later exposures. For instance, Günther et al. found an association between protein intake at 5–6 years of age and BMI. However, Beyerlein et al. only found an association of protein intake 4–5 years of age, not earlier (43). Rolland-Cachera also found an association between protein intake at 2 years of age and earlier BMI rebound, which, in turn, is related to higher adiposity (4, 51); however, this was not confirmed in a subsequent UK cohort (60). In the DONALD study, a high-protein intake was associated with higher BMI SDS at adiposity rebound in girls, but not in boys, but not with the timing of adiposity rebound (50).

We were also unable to separately assess any sex differences, although some studies reported stronger effects in boys than in girls (31, 49). This difference is potentially age dependent (39–41) due to different growth patterns or variations in the sensitivity to growth and sex hormones between sexes. Overall, the effect modification of age or gender on the effect of protein intake on excess weight or adiposity seems unclear and warrants further investigation in future studies.

### Public health relevance and implications

Although the effect size (0.06 kg/m^2^ higher BMI per E% from protein) in the meta-analysis could be regarded as small, such changes in mean BMI still shift the population distribution upwards and increase the chances of overweight and obesity (61). A previous individual-level meta-analysis of predictors of childhood obesity by Druet et al. (62) found that each one unit increase in weight SDS from 0 to 1 year of age was associated with a two-fold higher odds of childhood obesity and 23% higher odds of adult obesity. Weight gain from 0 to 2 years of age was also strongly and significantly related to a more than two-fold higher odds of childhood obesity in a subset. A rapid weight gain in especially the first year of life, adjusted for birth weight, is also associated with later overweight and/or obesity (63). Obesity in childhood and adolescence (7–18 years) is, in turn, strongly associated with having obesity in adulthood, although most adults with obesity did not have obesity in childhood (64). Evidently, overweight and obesity have negative consequences during childhood as well, including discrimination, stigma, musculoskeletal problems, and increased incidence of asthma. BMI in childhood and adolescence is also associated with the risk of cardiovascular disease (65, 66), type 2 diabetes, and mortality in adults (67).

### Strengths and limitations

In this work, we followed rigorous, state-of-the art methodologies for systematic reviews, with grading of the certainty of the evidence to help translating the findings into dietary guidelines. Limitations include the availability of few high-quality studies with comparable assessments, which prevented us from performing meta-analyses for several outcomes as well as from subgroup analyses, dose–response-meta-analysis, and the formal assessment of reporting bias. We were able to meta-analyze the association between E% from total protein and BMI in cohort studies, although the studies were small and had different risks of bias. Due to concomitant growth, defining adiposity in children based on BMI is complicated (68). This being said, BMI is a well-accepted reference for assessing overweight and obesity in large populations (69) and is strongly associated with body fat as measured by DEXA (70). According to Simmonds et al., BMI does have high sensitivity and specificity (and similar to waist circumference) for obesity in children, but less for overweight (71).

Additional limitations include the generally small sample sizes in the RCTs, which compromised statistical power and likely affected the ability to detect significant effects or changes in outcome variables. As to the observational studies, the dietary intake assessments are likely to some extent be affected by reporting errors, and the validity of the assessment methods were, with few exceptions (39, 40, 48), not reported. Many of the studies included in our analysis did not control protein intake for total energy intake, which is a limitation. Thus, higher protein intake could also be associated with higher total energy intake from other sources, albeit the studies in the meta-analysis were adjusted for energy intake (E% from protein). Moreover, few cohort studies with long follow-up assessed changes in protein intake over time. Theoretical dietary substitution effects, that is, replacing protein with other macronutrients or animal with plant protein, have been examined in a very few studies (39, 40, 42, 48) and were not evaluated separately.

## Conclusion

In this systematic review, we focused on healthy well-nourished children of Western populations below the age of 5 years. Based on consistent findings across cohort studies, it is *probable* that higher protein intake, in particular of animal origin, in children ≤18 months of age is linked to subsequent higher BMI. Limitations in the evidence were due to low availability of high-quality studies with comparable assessments and a lack of RCTs prevents higher evidence grading.

## Supplementary Material

Protein intake in children and growth and risk of overweight or obesity: A systematic review and meta-analysis

## Registration

PROSPERO registration number: CRD42021233197.
