# Supplementary material for: Protein intake in children and growth and risk of overweight or obesity: A systematic review and meta-analysis
Source: Food Nutr Res. 2022 Feb 21;66:10.29219/fnr.v66.8242. doi: 10.29219/fnr.v66.8242 (PMC8861858; doi:10.29219/fnr.v66.8242)
Supplement: Protein intake in children and growth and risk of overweight or obesity: A systematic review and meta-analysis [file FNR-66-8242-s001.docx]

# Supplementary material

# Search strategy

**Ovid MEDLINE(R) ALL 1946 to February 25, 2021**^1^

Date searched: 25 February, 2021

| 1 | Infant/ or Child, Preschool/ | 1252284 |
| --- | --- | --- |
| 2 | (infant? or Infancy or "early childhood" or toddler? or baby or babies).tw,kf. | 544615 |
| 3 | (child* adj2 (preschool or pre-school)).tw,kf. | 20986 |
| 4 | (("1" or "2" or "3" or "4" or "5") adj3 year? adj3 (age* or old*)).tw,kf. | 226842 |
| 5 | (("4" or "5" or "6" or "7" or "8" or "9" or "10" or "11" or "12" or "13" or "14" or "15" or "16" or "17" or "18" or "19" or "20" or "21" or "22" or "23" or "24") adj3 month? adj3 (age* or old*)).tw,kf. | 135016 |
| 6 | or/1-5 | 1671388 |
| 7 | exp Dietary Proteins/ | 100168 |
| 8 | (protein* adj3 (diet* or intake* or food or foods or feed or fed or consumption or consume* or nutrition or nutrient* or eat or eating or ate or animal? or fish or egg? or yolk? or fruit? or grain? or milk or dairy or meat? or nut? or pea or peas or poultry or bean? or soy? or whey or vegetable? or plant?)).tw,kf. | 93406 |
| 9 | or/7-8 | 161880 |
| 10 | Growth/ or exp Body Size/ or exp "Body Weights and Measures"/ or exp Body Composition/ | 687766 |
| 11 | (growth or height or weight or overweight or obesity or obese or "body mass index" or bmi or ((body or mass) adj2 (composition or fat or fat-free))).tw,kf. | 2693923 |
| 12 | or/10-11 | 2919342 |
| 13 | 6 and 9 and 12 | 4453 |
| 14 | 13 not (exp "Animals"/ not (exp "Animals"/ and "Humans"/)) | 3963 |

^1^ Abbreviations: tw: title or abstract kf: keyword. adj: adjacency.

**Embase Classic+Embase 1947 to 2021 February 25**^1^

Date searched: 26 February, 2021

| 1 | infant/ or baby/ or toddler/ or preschool child/ | 1147708 |
| --- | --- | --- |
| 2 | (infant? or Infancy or "early childhood" or toddler? or baby or babies).tw,kw. | 683081 |
| 3 | (child* adj2 (preschool or pre-school)).tw,kw. | 27321 |
| 4 | (("1" or "2" or "3" or "4" or "5") adj3 year? adj3 (age* or old*)).tw,kw. | 346402 |
| 5 | (("4" or "5" or "6" or "7" or "8" or "9" or "10" or "11" or "12" or "13" or "14" or "15" or "16" or "17" or "18" or "19" or "20" or "21" or "22" or "23" or "24") adj3 month? adj3 (age* or old*)).tw,kw. | 195479 |
| 6 | or/1-5 | 1767733 |
| 7 | protein intake/ | 45406 |
| 8 | (protein* adj3 (diet* or intake* or food or foods or feed or fed or consumption or consume* or nutrition or nutrient* or eat or eating or ate or animal? or fish or egg? or yolk? or fruit? or grain? or milk or dairy or meat? or nut? or pea or peas or poultry or bean? or soy? or whey or vegetable? or plant?)).tw,kw. | 117299 |
| 9 | or/7-8 | 136598 |
| 10 | exp body growth/ or body size/ or exp body weight/ or body height/ or body mass/ or exp body composition/ | 1050883 |
| 11 | (growth or height or weight or overweight or obesity or obese or "body mass index" or bmi or ((body or mass) adj2 (composition or fat or fat-free))).tw,kw. | 3634276 |
| 12 | or/10-11 | 3903220 |
| 13 | 6 and 9 and 12 | 5502 |
| 14 | 13 not ("animal"/ not "human"/) | 5355 |

^1^ Abbreviations: tw: title or abstract, kw: keyword, adj: adjacency.

**Cochrane Central Register of Controlled Trials**^1^

Date searched: 26 February, 2021

| #1 | ([mh ^Infant] OR [mh ^”Child, Preschool”]) | 37926 |
| --- | --- | --- |
| #2 | (infant OR infants OR infancy OR “early childhood” OR toddler OR toddlers OR baby OR babies):ti,ab,kw | 67519 |
| #3 | (child* NEAR/2 (preschool OR (pre NEXT school))):ti,ab,kw | 38478 |
| #4 | (((“1” OR “2” OR “3” OR “4” OR “5”) NEAR/3 year*) NEAR/3 (age* OR old*)):ti,ab,kw | 71794 |
| #5 | (((“4” OR “5” OR “6” OR “7” OR “8” OR “9” OR “10” OR “11” OR “12” OR “13” OR “14” OR “15” OR “16” OR “17” OR “18” OR “19” OR “20” OR “21” OR “22” OR “23” OR “24”) NEAR/3 month*) NEAR/3 (age* OR old*)) | 17348 |
| #6 | #1 OR #2 OR #3 OR #4 OR #5 | 151147 |
| #7 | [mh “Dietary Proteins”] | 4148 |
| #8 | (protein* NEAR/3 (diet* OR intake* OR food OR foods OR feed OR fed OR consumption OR consume* OR nutrition OR nutrient* OR eat OR eating OR ate OR animal OR animals OR fish OR egg OR eggs OR yolk OR yolks OR fruit* OR grain* OR milk OR dairy OR meat* OR nut OR nuts OR pea OR peas OR poultry OR bean* OR soy OR soybean* OR whey OR vegetable* OR plant OR plants)):ti,ab,kw | 11071 |
| #9 | #7 OR #8 | 11614 |
| #10 | ([mh ^Growth] OR [mh “Body Size”] OR [mh “Body Weights and Measures”] OR [mh “Body Composition”]) | 37537 |
| #11 | ((growth OR height OR weight OR overweight OR obesity OR obese OR “body mass index” OR bmi) OR ((body OR mass) NEAR/2 (composition OR fat OR (fat next free) ))):ti,ab,kw | 212513 |
| #12 | #10 or #11 | 213813 |
| #13 | #6 AND #9 AND #12 | 1385 |
| #14 | #6 AND #9 AND #12 in Trials | 1358 |

^1^Abbreviations: mh: subject heading. ti: title. ab: abstract. kw: keyword.

**Scopus**

Date searched: 26 February, 2021

Number of hits: 6805

TITLE-ABS-KEY(((infant* OR Infancy OR "early childhood" OR toddler* OR baby OR babies) OR (child* W/2 (preschool OR "pre-school")) OR ((("1" OR "2" OR "3" OR "4" OR "5") W/2 year*) W/2 (age* OR old*)) OR ((("4" OR "5" OR "6" OR "7" OR "8" OR "9" OR "10" OR "11" OR "12" OR "13" OR "14" OR "15" OR "16" OR "17" OR "18" OR "19" OR "20" OR "21" OR "22" OR "23" OR "24") W/2 month*) W/2 (age* OR old*))) AND (protein* W/2 (diet* OR intake* OR food OR foods OR feed OR fed OR consumption OR consume* OR nutrition OR nutrient* OR eat OR eating OR ate OR animal* OR fish OR egg* OR yolk* OR fruit* OR grain* OR milk OR dairy OR meat* OR nut* OR pea OR peas OR poultry OR bean* OR soy* OR whey OR vegetable* OR plant*)) AND ((growth OR height OR weight OR overweight OR obesity OR obese OR "body mass index" OR bmi) OR ((body OR mass) W/1 (composition OR fat OR "fat-free"))))

# Table S1. Excluded articles

| Reference | Reason for exclusion |
| --- | --- |
| Åkeson PM, et al. Growth and nutrient intake in three- to twelve-month-old infants fed human milk or formulas with varying protein concentrations. Journal of Pediatric Gastroenterology & Nutrition. 1998;26(1):1-8. | wrong exposure (formula) |
| Axelsson I, et al. Protein and energy intake during weaning: I. Effects on growth. Acta Paediatrica Scandinavica. 1987;76(2):321-7. | wrong exposure (formula) |
| Axelsson IE, et al. Formula with reduced protein content: effects on growth and protein metabolism during weaning. Pediatric Research. 1988;24(3):297-301. | wrong exposure (formula) |
| Axelsson IE, et al. Protein intake in early infancy: effects on plasma amino acid concentrations, insulin metabolism, and growth. Pediatric Research. 1989;26(6):614-7. | wrong exposure (formula) |
| Bates RD, et al. Milk and soy formulas: a comparative growth study. Annals of Allergy. 1968;26(11):577-83. | wrong age |
| Batra P, et al. Effects of two micronutrient-fortified food aid products containing different levels of dairy protein on anthropometric variables in rural pre-school children in Guinea-Bissau. FASEB Journal Conference: Experimental Biology. 2014;28(1). | wrong publication type (conference abstract) |
| Berkey CS, et al. Relation of childhood diet and body size to menarche and adolescent growth in girls. American Journal of Epidemiology. 2000;152(5):446-52. | wrong outcome (menarche, growth velocity) |
| Bhargava A. Protein and Micronutrient Intakes Are Associated with Child Growth and Morbidity from Infancy to Adulthood in the Philippines. Journal of Nutrition. 2016;146(1):133-41. | not relevant for Nordic population |
| Black AE, et al. The diets of preschool children in Newcastle upon Tyne, 1968-71. British Journal of Nutrition. 1976;35(1):105-13. | wrong outcome (protein intake) |
| Dorosty AR, et al. Factors associated with early adiposity rebound. ALSPAC Study Team. Pediatrics. 2000;105(5):1115-8. | wrong outcome (timing of rebound) |
| Fleddermann M, et al. Association of infant formula composition and anthropometry at 4 years: Follow-up of a randomized controlled trial (BeMIM study). PLoS ONE. 2018;13(7). | wrong age (1 month), wrong exposure |
| Fleddermann M, et al. Infant formula composition affects energetic efficiency for growth: the BeMIM study, a randomized controlled trial. Clinical Nutrition. 2014;33(4):588-95. | wrong age (1 month), wrong exposure |
| Gunther AL, et al. The influence of habitual protein intake in early childhood on BMI and age at adiposity rebound: results from the DONALD Study. International Journal of Obesity. 2006;30(7):1072-9. | wrong outcome (timing of rebound) |
| Hitchcock NE, et al. Dietary energy and nutrient intakes and growth of healthy Australian infants in the first year of life. Nutrition Research. 1982;2(1):13-9. | wrong outcome (protein intake) |
| Koletzko B, et al. Lower protein in infant formula is associated with lower weight up to age 2 y: a randomized clinical trial. American Journal of Clinical Nutrition. 2009;89(6):1836-45. | wrong age, wrong exposure (2 months, formula) |
| Liotto N, et al. Clinical evaluation of two different protein content formulas fed to full-term healthy infants: a randomized controlled trial. BMC Pediatrics. 2018;18(1):59. | wrong age (≤3 weeks) |
| Lonnerdal B, et al. Effects of formula protein level and ratio on infant growth, plasma amino acids and serum trace elements. II. Follow-up formula. Acta Paediatrica Scandinavica. 1990;79(3):266-73. | not relevant for Nordic population |
| Lonnerdal B, et al. Effects of formula protein level and ratio on infant growth, plasma amino acids and serum trace elements. I. Cow's milk formula. Acta Paediatrica Scandinavica. 1990;79(3):257-65. | wrong age (≤12 weeks) |
| Michaelsen KF. Nutrition and growth during infancy: The Copenhagen cohort study. Acta Paediatrica, International Journal of Paediatrics, Supplement. 1997;86(420):1-36. | wrong publication type |
| Morgan J, et al. Meat consumption is positively associated with psychomotor outcome in children up to 24 months of age. Journal of Pediatric Gastroenterology & Nutrition. 2004;39(5):493-8. | wrong exposure |
| Nicklas TA, et al. Dietary factors relate to cardiovascular risk factors in early life. Bogalusa Heart Study. Arteriosclerosis. 1988;8(2):193-9. | wrong outcome |
| Oropeza-Ceja LG, et al. Lower Protein Intake Supports Normal Growth of Full-Term Infants Fed Formula: A Randomized Controlled Trial. Nutrients. 2018;10(7):10. | wrong age (≤4 months) |
| Raiha N, et al. Milk protein intake in the term infant. I. Metabolic responses and effects on growth. Acta Paediatrica Scandinavica. 1986;75(6):881-6. | wrong age (newborn) |
| Raiha NC, et al. Whey predominant, whey modified infant formula with protein/energy ratio of 1.8 g/100 kcal: adequate and safe for term infants from birth to four months. Journal of Pediatric Gastroenterology & Nutrition. 2002;35(3):275-81. | wrong age (newborn) |
| Rogers I, et al. Milk as a food for growth? The insulin-like growth factors link. Public Health Nutrition. 2006;9(3):359-68. | wrong age (7-8 years) |
| Socha P, et al. Milk protein intake, the metabolic-endocrine response, and growth in infancy: data from a randomized clinical trial. American Journal of Clinical Nutrition. 2011;94(6):1776S-84S. | wrong age (≤8 weeks) |
| Tincu I. Influence of Protein Intake in the First Year of Life on Body Size and Igf-I Levels. Clinical Nutrition. 2019;38:S25-S6. | wrong publication type |
| Totzauer M, et al. Effect of Lower Versus Higher Protein Content in Infant Formula Through the First Year on Body Composition from 1 to 6 Years: Follow-Up of a Randomized Clinical Trial. Obesity. 2018;26(7):1203-10. | wrong age (≤2 months) |

# Supplementary figure S1


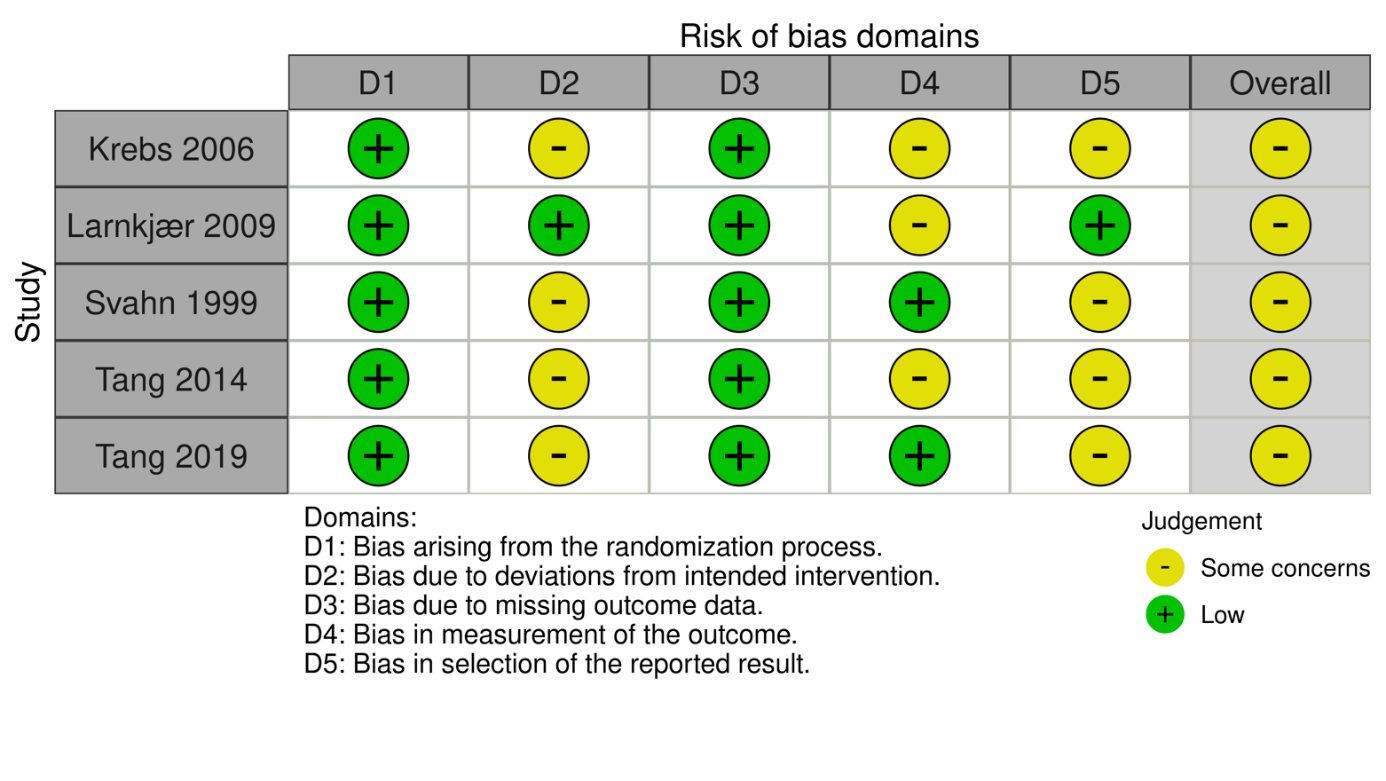
Risk of bias per domain in randomized controlled trials.

# Supplementary figure S2


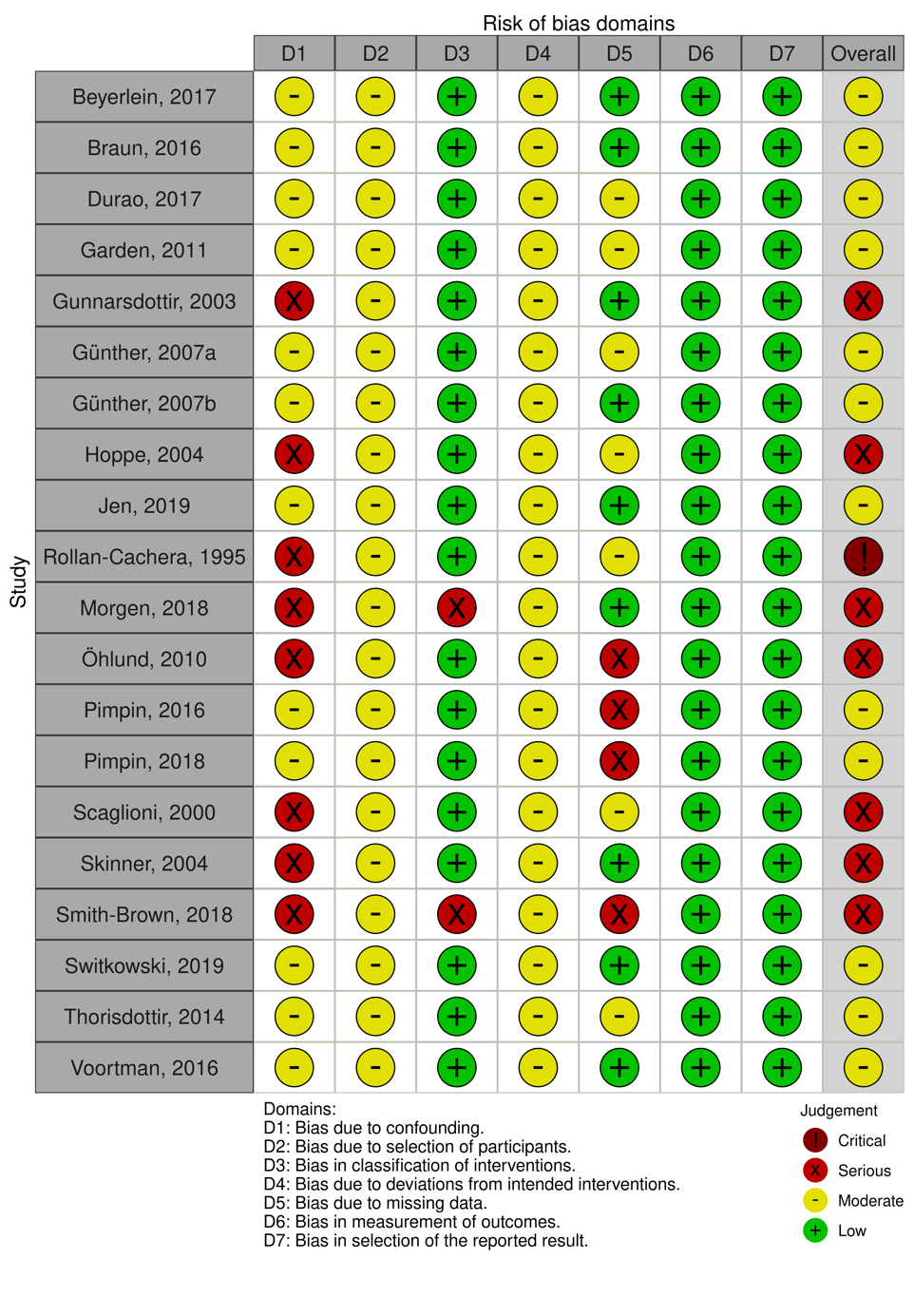
Risk of bias per domain in prospective cohort studies.
